# Feasibility of robotic-assisted surgery in advanced rectal cancer: a multicentre prospective phase II study (VITRUVIANO trial)

**DOI:** 10.1093/bjsopen/zrae048

**Published:** 2024-06-24

**Authors:** Atsushi Hamabe, Ichiro Takemasa, Masanori Kotake, Daisuke Nakano, Suguru Hasegawa, Akio Shiomi, Masakatsu Numata, Kazuhiro Sakamoto, Kei Kimura, Tsunekazu Hanai, Takeshi Naitoh, Yosuke Fukunaga, Yusuke Kinugasa, Jun Watanabe, Junichiro Kawamura, Mayumi Ozawa, Koji Okabayashi, Shuichiro Matoba, Yoshinao Takano, Mamoru Uemura, Yukihide Kanemitsu, Yoshiharu Sakai, Masahiko Watanabe

**Affiliations:** Department of Gastroenterological Surgery, Graduate School of Medicine, Osaka University, Osaka, Japan; Department of Surgery, Surgical Oncology and Science, Sapporo Medical University, Sapporo, Japan; Department of Surgery, Surgical Oncology and Science, Sapporo Medical University, Sapporo, Japan; Department of Surgery, Koseiren Takaoka Hospital, Takaoka, Japan; Department of Surgery, Tokyo Metropolitan Cancer and Infectious Diseases Center Komagome Hospital, Tokyo, Japan; Department of Gastroenterological Surgery, Faculty of Medicine, Fukuoka University, Fukuoka, Japan; Division of Colon and Rectal Surgery, Shizuoka Cancer Center Hospital, Nagaizumi, Japan; Department of Surgery, Yokohama City University, Yokohama, Japan; Department of Coloproctological Surgery, Juntendo University Faculty of Medicine, Tokyo, Japan; Division of Lower GI, Department of Gastroenterological Surgery, Hyogo Medical University, Nishinomiya, Japan; Department of Surgery, Fujita Health University, School of Medicine, Toyoake, Japan; Department of Lower Gastrointestinal Surgery, Kitasato University School of Medicine, Sagamihara, Japan; Gastroenterological Center, Department of Gastroenterological Surgery, Cancer Institute Hospital, Japanese Foundation for Cancer Research, Tokyo, Japan; Department of Gastrointestinal Surgery, Tokyo Medical and Dental University Graduate School of Medicine, Tokyo, Japan; Department of Surgery, Gastroenterological Center, Yokohama City University Medical Center, Yokohama, Japan; Department of Surgery, Kindai University Faculty of Medicine, Osakasayama, Japan; Department of Gastroenterological Surgery, Yokohama City University Graduate School of Medicine, Yokohama, Japan; Department of Surgery, Keio University School of Medicine, Tokyo, Japan; Department of Gastroenterological Surgery, Toranomon Hospital, Tokyo, Japan; Department of Surgery, Southern TOHOKU Research Institute for Neuroscience, Southern TOHOKU General Hospital, Koriyama, Japan; Department of Gastroenterological Surgery, Graduate School of Medicine, Osaka University, Osaka, Japan; Department of Colorectal Surgery, National Cancer Center Hospital, Tokyo, Japan; Department of Surgery, Osaka Red-Cross Hospital, Osaka, Japan; Department of Surgery, Kitasato University Kitasato Institute Hospital, Tokyo Japan

## Abstract

**Background:**

The potential benefits of robotic-assisted compared with laparoscopic surgery for locally advanced cancer have not been sufficiently proven by prospective studies. One factor is speculated to be the lack of strict surgeon criteria. The aim of this study was to assess outcomes for robotic surgery in patients with locally advanced rectal cancer with strict surgeon experience criteria.

**Methods:**

A criterion was set requiring surgeons to have performed more than 40 robotically assisted operations for rectal cancer. Between March 2020 and May 2022, patients with rectal cancer (distance from the anal verge of 12 cm or less, cT2–T4a, cN0–N3, cM0, or cT1–T4a, cN1–N3, cM0) were registered. The primary endpoint was the rate positive circumferential resection margin (CRM) from the pathological specimen. Secondary endpoints were surgical outcomes, pathological results, postoperative complications, and longterm outcomes.

**Results:**

Of the 321 registered patients, 303 were analysed, excluding 18 that were ineligible. At diagnosis: stage I (*n* = 68), stage II (*n* = 84) and stage III (*n* = 151). Neoadjuvant therapy was used in 56 patients. There were no conversions to open surgery. The median console time to rectal resection was 170 min, and the median blood loss was 5 ml. Fourteen patients had a positive CRM (4.6%). Grade III-IV postoperative complications were observed in 13 patients (4.3%).

**Conclusion:**

Robotic-assisted surgery is feasible for locally advanced rectal cancer when strict surgeon criteria are used.

## Teaser

We are the first in Japan to conduct the multicentre prospective VITRUVIANO trial to evaluate the oncological utility of robotic surgery for advanced rectal cancer. In this article, we report the results of this analysis. In this trial, by setting stricter surgical qualifications compared with those of past clinical trials, we were able to control the quality of the surgery and, as a result, demonstrate favourable outcomes.

## Introduction

In recent years, the number of robotic-assisted operations for rectal cancer has increased globally. However, there is insufficient prospective data proving its effectiveness compared with conventional laparoscopic surgery. The ROLARR trial, reported in 2017, was an RCT that compared robotic and laparoscopic surgery, with the conversion rate to open surgery as the primary endpoint. It failed to demonstrate the superiority of robotic surgery or establish its effectiveness in terms of the secondary endpoint of positive circumferential resection margin (CRM) rate^1^. Although this trial might suggest that robotic surgery does not provide additional value compared with laparoscopic surgery, the ROLARR trial did not stringently define surgical proficiency in robotic surgery. This lack of definition may have led to the inclusion of surgeons less experienced with robotic surgery and may have contributed to the failure to demonstrate the superiority of robotic surgery.

The Japanese multicentre prospective study investigating laparoscopic surgery for locally advanced rectal cancer with evaluation of CRM and TME quality (PRODUCT trial), examined the treatment outcomes of minimally invasive surgery for stage II or III locally advanced rectal cancer^2^. As a result, the positive CRM rate, a primary endpoint of the PRODUCT trial, was 8.6%. In the PRODUCT trial, 45% of the registered patients had robotically assisted operations, with no strict regulations regarding surgeon experience.

The aim of this study was to investigate treatment outcomes of robotic surgery under conditions with strictly defined proficiency in robotic surgery.

## Methods

### Patients

The selection criteria were patients diagnosed with rectal cancer (excluding squamous cell carcinoma and neuroendocrine tumour) through endoscopic biopsy of the rectal primary lesion; suitability for curative resection based on preoperative examination and imaging diagnosis; tumour lower edge being within 12 cm from the anal verge with clinical staging of cT2–T4a, cN0–N3, cM0, or cT1–T4a, cN1–N3, cM0; ineligibility for endoscopic removal; age 20 years or older; and being scheduled for robotic surgery for rectal resection.

The exclusion criteria included tumour depth of cT4b, multiple cancers (either colorectal or different primary sites) and patients judged to lack the capacity to consent. Rectal MRI was used for the assessment of T and N staging, and the clinical stage was assessed based on the images before neoadjuvant treatment. This study is a multicentre prospective observational study conducted by 24 facilities affiliated with the Japan Society of Laparoscopic Colorectal Surgery. The indications for preoperative treatment and lateral lymph node dissection were decided at each institution’s discretion. The target number of patients was set to 300, referencing the 303 patients analysed in the previous PRODUCT trial. The study protocol was approved by the ethics committees of the participating institutions, and consent forms were obtained from all participants. This study was registered in the University Hospital Medical Information Network (UMIN) Clinical Trials Registry, Identification Number UMIN000039685.

### Surgeon criteria

Previous reports have indicated that mastering the learning curve for robotic-assisted rectal resection surgery requires approximately 30 patients^3,4^. In this study, given the importance of quality control in surgery, the authors set more stringent criteria for the surgeon, limiting the study to surgeons who have performed more than 40 robotically assisted operations. At the time of this study, to qualify as a robotic surgeon in Japan, surgeons needed to be a certified physician through the Endoscopic Surgical Skill Qualification System of the Japan Endoscopic Surgery Association, which includes a video review^5^. Therefore, in this study, a new video review was not conducted but instead set the criteria for surgeons based on the number of robotic operations they had performed.

### Neoadjuvant treatment

According to the Japanese guideline, the standard treatment for locally advanced rectal cancer is upfront surgery followed by adjuvant chemotherapy^6^. For rectal cancer with a high risk of local recurrence, preoperative chemoradiotherapy (CRT) is recommended, although a detailed description of the indication for CRT is lacking. In this study, the indication for neoadjuvant treatment is determined by each facility. After the completion of CRT, surgery is recommended to be performed within 8–12 weeks. The registration of patients that underwent neoadjuvant chemotherapy or TNT (total neoadjuvant therapy) was also permitted.

### Transanal approach

At the start of the study, following discussions on the use of the transanal approach, transanal total mesorectal excision (TaTME) was considered to be utilized as an adjunct to robotic procedures. It was agreed that TaTME would only be used to create a ‘receiving space’ for robotic dissection operations caudal to the pelvic splanchnic nerves S4, without performing dissection in the cranial direction.

### Endpoints

The positive CRM rate, an oncological indicator for rectal cancer surgery^7–9^, was set as the primary endpoint. The CRM was measured using a semi-opened circular specimen processing method adopted in the previous PRODUCT trial^2,10,11^. Briefly, the area of the rectum between 2 cm above and below the borders of the rectal cancer is not incised and the corresponding mesorectum is left attached to the rectum to measure the CRM. In assessing the CRM, if the tumour invasion in lymph node metastasis, extramural vascular invasion (EMVI) or tumour deposit is closer to the dissected plane than the main tumour invasion, the closer distance is recorded as the CRM. CRM was considered positive if it was 1 mm or less. Secondary endpoints included the completeness of total mesorectal excision (TME), successful resection rate, open conversion rate, intraoperative adverse event incidence rate, surgical outcomes, short-term postoperative outcomes (recovery of bowel movement, postoperative hospital stay, postoperative morbidity/surgery-related mortality rate, reoperation rate), urinary function and long-term postoperative outcomes (3-year local recurrence-free survival rate, disease-free survival rate and overall survival rate). The quality of the mesorectal excision was categorized as complete, nearly complete or incomplete according to the Dutch TME trial criteria^12^.

### Definition of urinary dysfunction

Early urinary dysfunction was defined according to the previous definition used in the JCOG0212 trial^13^. Briefly, urinary dysfunction post-surgery was determined by measuring residual urine volume, typically three times within 10–14 days post-surgery. If the total of discharged and residual urine exceeded 150 ml, it counted as one measurement. Dysfunction was defined as either having ≥50 ml residual urine in one or more measurements when measured more than three times or, when measured twice, it was defined by ≥50 ml residual urine or a clinical diagnosis of urinary dysfunction.

### Statistical analysis

Results are expressed as the number of patients evaluated for categorical data, or as the median and i.q.r. for quantitative data. Univariate analyses were performed using Fisher’s exact test.

## Results

### Patient background and tumour characteristics

**Fig. 1:**
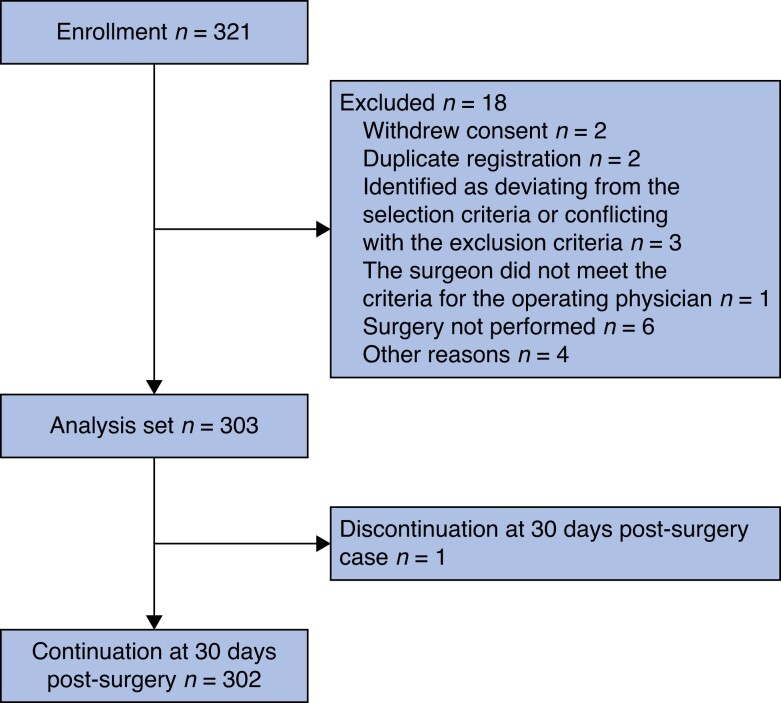
Flow of patient inclusion in the VITRUVIANO trial

A total of 321 patients were registered between March 2020 and May 2022 (*Fig. 1*). Eighteen patients were excluded: two withdrew consent, two were duplicate registrations, three deviated from the selection criteria or met exclusion criteria, one involved surgery by a non-compliant surgeon, six did not undergo surgery and four were excluded for other reasons. Ultimately, 303 patients were analysed for the primary endpoint.

*Table 1* details patient and tumour characteristics. The median age was 67 years, with males accounting for 65% of the population. The median distance from the anal verge (AV distance) was 7.0 cm, with the stage distribution being 68 patients (22.4%) at stage I, 84 (27.7%) at stage II and 151 (49.8%) at stage III. There were 25 patients with lateral lymph node metastasis (8.3% at clinical diagnosis). Of the 292 patients that underwent MRI at diagnosis, seven had a distance of 1 mm or less from the deepest part of the tumour to the mesorectal fascia. *Table 2* outlines the preoperative treatment: surgery was the first approach in 81.5% of patients, with the remaining 18.5% receiving neoadjuvant treatment. Among the 56 patients who received neoadjuvant treatment, two (one each from CRT and TNT) achieved a clinical complete response.

**Table 1:** Patient and tumour characteristics

**Patient characteristics (*n* = 303)**	
Age (years), median (i.q.r.)	67 (56–73)
** Sex**	
Male	197 (65.0)
Female	106 (35.0)
BMI (kg/m^2^), median (i.q.r.)	23.0 (20.8–25.5)
** ASA-PS**	
1–2	291 (96.0)
3	12 (4.0)
**Tumour characteristics at initial examination**	
Distance from the anal verge (cm), median (i.q.r.)	7.0 (5.0–10.0)
Distance from the anal verge in patients using TaTME (cm), median (i.q.r.)	5.5 (4.0–6.0)
** Location of the centre of the tumour**	
Anterior	84 (27.7)
Posterior	77 (25.4)
Right	52 (17.2)
Left	55 (18.2)
Circular	35 (11.6)
** Tumour stage at baseline**	
cT1	3 (1.0)
cT2	82 (27.1)
cT3	187 (61.7)
cT4a	31 (10.2)
** Nodal status at baseline**	
cN0	152 (50.2)
cN1	106 (35.0)
cN2	25 (8.3)
cN3	20 (6.6)
Suspected metastasis to lateral lymph node	25 (8.3)
** Clinical stage at baseline**	
Stage I	68 (22.4)
Stage II	84 (27.7)
Stage III	151 (49.8)
Predicted CRM from mesorectal fascia on MRI (mm), median (i.q.r.) (*n* = 292)*	5.0 (3.0–9.3)
Positive (≤1 mm)	7 (2.4)
Negative (>1 mm)	285 (97.6)

Values are *n* (%) unless otherwise stated.

^*^Excluding 11 patients without MRI. ASA-PS, American Society of Anesthesiologists physical status; CRM, circumferential resection margin; TaTME, transanal total mesorectal excision; i.q.r., interquartile range.

**Table 2:** Neoadjuvant treatment

**Neoadjuvant treatment**	
None	247 (81.5)
(Chemo)radiotherapy	17 (5.6)
Neoadjuvant chemotherapy	29 (9.6)
Total neoadjuvant therapy	10 (3.3)
**Tumour characteristics after neoadjuvant therapy (*n* = 56)**	
** Tumour stage after neoadjuvant therapy**	
ycT0	2 (3.6)
ycT1	0 (0.0)
ycT2	6 (10.7)
ycT3	45 (80.4)
ycT4a	3 (5.4)
** Nodal status after neoadjuvant therapy**	
ycN0	38 (67.9)
ycN1	8 (14.3)
ycN2	2 (3.6)
ycN3	8 (14.3)

Values are *n* (%).

### Operative results

*Table 3* highlights surgical outcomes. Rectal transection could be carried out transabdominally in over 80% of the patients, whereas TaTME was also used in 35 patients (11.6%). Of the 269 patients that had the anus preserved, 134 had a diverting stoma created. One patient had a combined resection of the seminal vesicle, and another required a combined resection of the bladder. Lateral lymph node dissection was performed in 70 patients (23.1%). The median console time for rectal resection was 170 min, and the median total operation time was 313 min. In the patients excluding those that underwent lateral lymph node dissection and TaTME, the median console time and total operation time were 178 and 282 min, respectively. The median blood loss was 5 ml. The open conversion rate was 0%.

**Table 3:** Operative results

**Surgical procedure**	
LAR	256 (84.5)
ISR	13 (4.3)
APR	31 (10.2)
Hartmann	3 (1.0)
**TaTME**	
Yes	35 (11.6)
No	268 (88.4)
Creation of diverting stoma (*n* = 269)*	
Yes	134 (49.8)
No	135 (50.2)
**Combined resection**	
Seminal vesicle	1 (0.3)
Bladder	1 (0.3)
**LLND**	
Yes	70 (23.1)
No	233 (76.9)
**Operative duration (min), median (i.q.r.)**	
Console time to rectal resection	170 (131–214)
Total operative time	313 (249–434)
**Operative duration (min) excluding patients of LLND and TaTME, median (i.q.r.) (*n* = 214)**	
Console time to rectal resection	178 (139–213)
Total operative time	282 (239–356)
Blood loss (ml), median (i.q.r.)	5 (0–28)
**Intraoperative morbidity**	
Vascular injury	4 (1.3)
Organ injury	3 (1.0)
Conversion to open surgery	0 (0.0)

Values are *n* (%) unless otherwise stated.

^*^The patients in which the anus was preserved. APR, abdominoperineal resection; AR, anterior resection; ISR, intersphincteric resection; LAR, low anterior resection; LLND, lateral lymph node dissection; TaTME, transanal total mesorectal excision; i.q.r., interquartile range.

### Pathological results

*Table 4* provides the pathological results. The median CRM, the primary endpoint, was 6.0 mm, with 14 positive patients, resulting in a CRM positivity rate of 4.6%. Of these 14 patients, 11 were CRM-positive at the deepest part of the main tumour, two were CRM-positive at tumour deposits and one was CRM-positive at a lymph node. Excluding the 68 patients diagnosed as stage I at diagnosis, the positive CRM rate was found to be 6.0% among those in stages II and III. For the quality assessment of TME, 98.4% were rated as complete. For the pathological stage, there were eight patients (2.6%) of pathological complete response, one stage 0 (0.3%), 109 stage I (36.0%), 82 stage II (27.1%), 99 stage III (32.7%) and four (1.3%) diagnosed with distant metastasis during surgery (stage IV). In the 56 patients that received neoadjuvant treatment, the pathological complete response (pCR) rate was 14.3%. In patients that received CRT and TNT, the pCR rate increase was 22.2%. Of the 70 patients where lateral lymph node dissection was performed, 13 were pathologically confirmed to be metastasis positive. The results of the analysis of risk factors for CRM positivity are presented in *Table 5*. Sex, BMI, neoadjuvant treatment, distance from the anal verge and lymph node metastasis were not significantly correlated with the rate of CRM positivity. However, patients with a pathological tumour stage of ≥T3 had a significantly higher rate of CRM positivity compared with patients staged ≤ T2 (*P* = 0.0001).

**Table 4:** Pathological results

**CRM (mm), median (i.q.r.)**	6.0 (3.3–10.0)
Positive	14 (4.6)
Negative	289 (95.4)
**CRM excluding patients using TaTME (mm), median (i.q.r.), (*n* = 268)**	6.0 (3.0–10.0)
Positive	14 (5.2)
Negative	254 (94.8)
**Tumoural region with CRM positivity (*n* = 14)**	
Main tumour	11 (3.6)
Tumour deposit	2 (0.7)
Lymph node	1 (0.3)
**Total mesorectal excision**	
Complete	298 (98.4)
Nearly complete	3 (1.0)
Incomplete	2 (0.7)
**Histological type**	
Adenocarcinoma	294 (97.0)
Mucinous	9 (3.0)
**pT**	
pT0	8 (2.6)
pTis	1 (0.3)
pT1	36 (11.9)
pT2	95 (31.4)
pT3a, b	116 (38.3)
pT3c, d	33 (10.9)
pT4a	13 (4.3)
pT4b	1 (0.3)
pN*	
N0	203 (67.0)
N1	72 (23.8)
N2	18 (5.9)
N3	10 (3.3)
Metastasis to lateral lymph node (*n* = 70)*	13 (18.5)
**pM**	
M0	299 (98.7)
M1	4 (1.3)
**Proximal resection margin (mm), median (i.q.r.)**	
Negative	303 (100)
Positive	0 (0)
**Distal resection margin (mm), median (i.q.r.)**	
Negative	303 (100)
Positive	0 (0)
**Pathological stage**	
pCR	8 (2.6)†
Stage 0	1 (0.3)
Stage I	109 (36.0)
Stage II	82 (27.1)
Stage III	99 (32.7)
Stage IV	4 (1.3)
pCR rate (%)	14.3
Negative circumferential and distal margins and complete total mesorectal excision	284 (93.7)

Values are *n* (%) unless otherwise stated.

^*^Patients who underwent LLND (lateral lymph node dissection). †In the 56 patients of neoadjuvant treatment, the pCR rate was 14.3%. CR, complete response, CRM, circumferential resection margin; TaTME, transanal total mesorectal excision; i.q.r., interquartile range.

**Table 5:** Univariate analyses of the risk factors for positive CRM

	CRM negative (*n* = 289)	CRM positive (*n* = 14)	CRM positive rate	*P*
**Sex**				0.5715
Male	189	8	4.1%	
Female	100	6	5.7%	
**BMI**				0.1656
<24 kg/m^2^	166	11	6.2%	
≥24 kg/m^2^	123	3	2.4%	
**Neoadjuvant treatment**				0.4794
Yes	55	1	1.8%	
No	234	13	5.3%	
**Distance from the anal verge**				0.7649
≤5 cm	80	3	3.6%	
>5 cm	209	11	5.0%	
**Pathological tumour stage**				0.0001
≤T2	140	0	0%	
≥T3	149	14	8.6%	
**Pathological nodal stage**				0.5623
N0	194	8	4.0%	
N1–3	95	6	5.9%	

CRM, circumferential resection margin.

### Postoperative outcomes

*Table 6* presents the postoperative outcomes. The median time to first postoperative flatus was 1 day, the median time to first bowel movement was 2 days, and the median time to commence food intake was 3 days. The median postoperative hospital stay was 13 days. The incidence rate of grade III or IV complications was 6.3%, with anastomotic leakage occurring in five patients (1.7%). The reoperation rate within 30 days post-surgery was 2.0%, and the mortality rate was 0%. The incidence of early postoperative urinary dysfunction was 38.6% overall.

**Table 6:** Postoperative results

**Postoperative recovery (days), median (i.q.r.)**	
Time to first defaecation	2 (1–3)
Time to commence food intake	3 (3–5)
Postoperative hospital stay	13 (9–18)
**Grade III–IV postoperative complications (including duplicates)**	19 (6.3)
Anastomotic leakage	5 (1.7)
Infection	6 (2.0)
Ileus	5 (1.7)
Stoma outlet obstruction	1 (0.3)
Anastomotic bleeding	1 (0.3)
Cholecystitis	1 (0.3)
Cholangitis	1 (0.3)
Reoperation within 30 days post-surgery	6 (2.0)
30-day mortality	0 (0.0)

Values are *n* (%) unless otherwise stated.

## Discussion

The VITRUVIANO trial demonstrated that robotic surgery for locally advanced rectal cancer was feasible in terms of the CRM positivity rate, a vital oncological factor in rectal cancer surgery. This study only included patients being operated on by highly skilled surgeons. It suggests that to perform robotic surgery safely for advanced rectal cancer requires a robust educational system for training. The PRODUCT trial included patients with stage II or III at diagnosis and involved different participating facilities^2^. Therefore, it is not possible to directly compare its results with this study, although the 4.6% CRM positivity rate in the present study was lower than the 8.6% observed in the PRODUCT trial. In the VITRUVIANO trial, the CRM positivity rate was 6.0%, even when the analysis was confined to stage II or III patients, suggesting a favourable outcome. It is surmised that surgeons with significant experience and advanced technical skills could optimally utilize various features of robotic surgery, such as the precise motion of surgical instruments or multijoint functions, during the procedure. This allowed them to operate along the appropriate surgical dissection plane based on preoperative diagnoses to achieve sufficient CRM. Recent literature also suggests that robotic surgery could offer advantages in this regard, provided the surgeon has the necessary technical skills to perform a complete good oncologic resection^14^.

There have been only a few RCTs comparing robotic surgery with laparoscopic surgery^15–18^. The ROLARR trial indicated that robotic surgery did not offer significant additional benefits over laparoscopic surgery^1^. Similarly, two RCTs from South Korea found no significant difference in CRM between robotic and laparoscopic surgery^19,20^. Although both studies were conducted by surgeons with sufficient experience, they were carried out in the early 2010s. One was a randomized phase II study with a limited number of patients and the other was prematurely halted due to registration issues. There have been few observational studies regarding whether robotic surgery reduces CRM positivity^21^. The REAL trial is the first phase III study demonstrating that robotic surgery can improve the rate for positive CRM, a surrogate marker for long-term prognosis, in comparison with laparoscopic surgery for rectal cancer patients with an AV of 10 cm or less, of cT1–T3 N0–N1, or ycT1–T3 NX^22^. The short-term results, reported in 2022, indicated a CRM positivity rate for robotic surgery of 4.0%, significantly better than the 7.2% for laparoscopic surgery, thereby demonstrating the oncological superiority of robotic surgery for rectal cancer. In Japan, the standard treatment for rectal cancer is a surgery-first approach; therefore, the proportion of patients receiving neoadjuvant treatment in the VITRUVIANO trial was only 20%, less than the approximately 43% in the REAL trial. However, the distribution of pathological stages was nearly identical. Both the VITRUVIANO and REAL trials were conducted in Asia and included patients with similar BMI (approximately 23). In the REAL trial, the CRM positivity rates for robotic and laparoscopic surgeries were 4.0% and 7.2% respectively, whereas in the VITRUVIANO and PRODUCT trials, the rates were 4.6% and 8.6% respectively. These data suggest that even when surgeons with significant surgical experience perform rectal resection using laparoscopy, there exists a lower threshold for the rate of positive CRM. However, by utilizing a robotic approach, they can carry out higher-quality surgery, likely due to the unique advantages of robotics, leading to an even lower rate of positive CRM. Given that the safety of robotic surgery has already been validated by several studies, including metadata analysis^23^, the fact that robotic surgery can add oncological benefits should significantly strengthen its usefulness.

The REAL trial demonstrated that in patients with high difficulty factors such as male sex, proximity to the anus, and advanced depth of invasion, robotic surgery can mitigate the challenges associated with laparoscopic surgery. However, it also revealed that the rate of CRM positivity is greater in male patients than in female patients, and greater in lower rectal cancers than in higher rectal cancers, indicating that some patients are difficult even when robotic surgery is employed. Nonetheless, in the VITRUVIANO trial, the CRM positivity rate was not high in men or in lower rectal cancer patients. It has been suggested that under the trial’s conditions, which included preoperative MRI for depth-of-invasion diagnosis and stringent criteria for the surgeon, the difficulty could be mitigated. Although it was impossible in that trial to completely avoid the impact of tumour depth, which directly affects CRM measurement, it is believed that the use of robotics allowed for precise dissection at the intended layer even in the narrow space of the pelvis, depending on tumour depth.

This study demonstrated low complication rates. In the PRODUCT trial and other prior trials conducted in Japan, such as the multicentre prospective JCOG0212 trial comparing TME with TME plus lateral lymph node dissection by open surgery, and the other trial verifying the feasibility of laparoscopic rectal resection for early rectal cancer, the incidence of grade III or higher complications in rectal cancer surgery was about 10–20%^24,25^. Internationally, the COREAN, COLOR II, ALaCaRT and Z6051 trials all demonstrated similar complication rates, emphasizing the importance of reducing complication rates in rectal cancer surgery^26–29^. In the current study, the incidence of grade III or higher complications was notably low at 6.3%, significantly lower than the rates shown in prior studies. In the REAL trial, the incidence of grade III or higher complications in robotic surgery was 3.0%, which was superior to those in laparoscopic surgery^22^. Considering these findings, the authors conclude that robotic surgery carried out under strict surgeon qualifications can be effective not only for improving oncological treatment outcomes but also for enhancing safety. In terms of urinary dysfunction, as the criterion in this trial based on that of JCOG0212 was strict, the rate of urinary dysfunction was found to be high^13^.

Historically in Japan, the mesorectum has been excised from the rectum according to the Japanese Society for Cancer of the Colon and Rectum guidelines for colorectal cancer treatment^6^. Lymph nodes have been inspected, and the gross morphology of the tumour has been evaluated or a fresh sample of the tumour has been harvested by opening the rectal wall and exposing the lumen. However, this method does not allow for CRM measurement. It has been nearly four decades since the importance of the CRM concept was recognized^30,31^. However, recent clinical studies, in the context of advancements in preoperative treatment and minimally invasive procedures, have reaffirmed the importance of assessing CRM as an indicator of radicality in rectal cancer surgery^8,9^. Amid this, in Japan we have incorporated a semi-opened circular specimen processing method into clinical practice, and it has been used to evaluate the CRM in preceding studies, including the PRODUCT trial^2,10,11^.

This study has several limitations. First, the study is not a comparative study but a single-group prospective trial; therefore, it does not lend itself to direct comparison with other studies, including the PRODUCT trial. Second, as no long-term prognosis analysis has been conducted, the oncological impact of robotic surgery cannot yet be determined. Long-term oncological outcome will be published in a future analysis with a mature data set.

## Data Availability

The data sets used during the current study are available from the corresponding author on reasonable request.
